# Impaired brain intrinsic connectivity in long COVID during cognitive exertion revealed by independent component analysis

**DOI:** 10.1038/s41598-026-36986-1

**Published:** 2026-02-09

**Authors:** Leighton Barnden, James Baraniuk, Maira Inderyas, Natalie Eaton-Fitch, Sonya Marshall-Gradisnik, Kiran Thapaliya

**Affiliations:** 1 National Centre for Neuroimmunology and Emerging Diseases, Griffith University, Gold Coast, QLD Australia; 2 Department of Medicine, Georgetown University, Washington, DC USA

**Keywords:** fMRI connectivity, Long COVID, Stroop interference, ICA, Inter-network, Cognitive fatigue, Compensatory, Neuroscience, Diseases, Medical research

## Abstract

Cognitive dysfunction is a symptom of Long COVID. To characterize functional connectivity changes that may contribute to cognitive dysfunction in Long COVID (LCov), two consecutive 450 s functional magnetic resonance imaging (fMRI) scans (Runs 1 and 2) were acquired on a 7 Tesla MRI scanner. During both, the Stroop colour-word task engaged intrinsic brain networks for conflict detection, conflict resolution and response execution. In this exploratory study we acquired data from 19 LCov and 16 healthy control (HC) participants. The aggregate dataset was subjected to independent component analysis (ICA) for each run to isolate 15 components with distinct spatial and temporal signatures. For each component we tested (1) for differences between LCov and HC (inter-network) connectivity to the rest of the brain and (2) for correlation of LCov connectivity with illness duration. Stroop response times (RTs) were slower in LCov than in HC in both Runs (*p* = 0.001, 0.003). Each ICA component occupied the hubs of a known intrinsic network. LCov had different inter-network connectivity to HC for Salience, Language, Central Executive, Sensorimotor and Visual networks. LCov deficits in Salience inter-network connectivity in Run 2 were deeper and more widespread than in Run 1. In contrast, Run 2 connectivity was *greater* in LCov for the angular gyrus which integrates visual, motor and language inputs and responses. With longer illness duration, LCov connectivity weakened to critical networks but showed compensatory effects in Lingual gyri. Slower Stroop RTs in both Runs, and Salience, Language and Central Executive inter-network connectivity deficits, and illness duration dependence, support cognitive impairment in LCov with some compensatory connectivity increases.

## Introduction

A study of adults who had been infected with the Sars2 COVID-19 Severe Acute Respiratory Syndrome Coronavirus 2 (SARS-CoV-2) virus found 14% had continuing symptoms that met long COVID (LCov) criteria^1^. “Brain fog” and cognitive dysfunction are important disabling elements of LCov^2–4^. Improved understanding of their underlying brain pathology may lead to development of new therapies^5^. Impairment of executive function, memory, attention, processing speed and other cognitive domains occur in 22–78% of patients with mild to moderate LCov^6–10^. Resting state functional connectivity is consistently altered in LCov^11–13^.

Widespread abnormalities in gray matter volumes, white matter integrity or cerebral perfusion have also been documented^14–16^. The virus has been found to act as a fusogen that disrupts neuronal function throughout the brain^17^.

COVID-19 has a broad impact on the functional connectome characterised by weaker functional connectivity, altered patterns of information processing efficiency and effective information flow^18^. Functional MRI (fMRI) recorded during cognitive provocation with the Stroop interference task found stronger functional connectivity in LCov for the left brainstem rostral medulla to the midbrain right cuneiform nucleus and right lateral parietal hub of the default mode network. These regions were also correlated with clinical scores for disability and autonomic function^19^. Other connections between network hubs were stronger in healthy controls (HC) than LCov. Brainstem subregion volumes were larger in LCov than controls^20^.

Brain networks with correlated activity have been identified that will be involved in cognitive dysfunction^21^. The default mode network (DMN), salience (SA) network and central executive network (CE also known as frontoparietal) are regarded as ‘core’ networks^22^ with the salience network activating only one depending whether stimuli are internal (DMN) or external (CE)^23^. Other networks include the sensorimotor, language, visual, dorsal attention and cerebellar.

In addition to these *intra*-network connections, intrinsic *inter*-network connections have been recognised. Inter-network connectivity has been shown to be age-dependent^24^ and abnormal in schizophrenia^25^, late-life depression^26^ and brain tumour subjects^27^. Previous studies utilised structural MRI of network hubs^24^ and resting-state functional connectivity^25^ and were enhanced by independent component analysis^26^. Inter-network connectivity has not been investigated in LCov and was the primary motivation for this analysis.

To examine details of cognitive dysfunction we analyzed fMRI acquired during the Stroop colour-word interference task^28^. Such neuropsychological interference tasks provoke prolongation of response times when irrelevant features of the task “interfere” with task-relevant elements^4,29^. Network processes invoked by the Stroop task include selective attention for task execution by the dorsolateral prefrontal cortex (DLPFC) of the CE network^30,31^. The lateral prefrontal cortex is proposed to resolve the colour – word interference of the Stroop task^32,33^ while the posterior DLPFC creates the appropriate rules to complete the task^34^. The left and right DLPFC monitor task conflict and adaptation^35^. The anterior cingulate cortex (ACC) serves for error checking by monitoring bilateral DLPFC function^34,36–38^. The posterior dorsal ACC activates the medial supplementary motor area leading to the ultimate button press that marks the end of each test^35^. Processing of the difficult incongruent stimulus activates the left intraparietal lobe and bilateral extrastriate visual cortex^39^. Distracting stimuli may stimulate the right ventral attention network with activation of the right temporal parietal junction, inferior frontal gyrus and insula^40,41^. The right anterior insula is closely connected to the dorsal ACC (dACC) within the salience network that can recognise the interference of the incongruent condition and recruit inhibitory control mechanisms through the dACC, supplemental motor area (SMA) and separate connections to the right putamen to facilitate planning for flexible goal-oriented, adaptive behavior^42,43^. Resting state fMRI would not engage these cognitive exertion networks.

Based on the functional connectivity for the Stroop task in HC and alterations in functional connectivity in LCov^19^, we predicted that LCov would have altered inter-network connectivity when quantified using highly specific independent component analysis (ICA). Two fMRI runs separated by 90 s were acquired in order to explore connectivity changes associated with cognitive fatigue, conflict adaption and practice effects. We hypothesized that significant differences in inter-network connectivity during the Stroop task at baseline would establish the baseline for cognitive dysfunction. Comparison of the first and second runs may then provide insights into fatigue, and adaptive or practice effects in LCov.

## Methods

### Subjects

The study was approved by Griffith University Human Research Ethics Committee (2022/666) and conducted in accordance with the relevant guidelines and regulations under the Helsinki Declaration. Written informed consent was obtained from all individuals. This cross-sectional investigation was conducted at the National Centre for Neuroimmunology and Emerging Diseases (NCNED) on the Gold Coast, Queensland, Australia. Eligible participants were medical-practitioner referred and assessed using the NCNED research questionnaire for LCov post-infection duration of illness. For fatigue affected subjects, we recorded severity of post-exertional malaise, cognitive disturbances, immune manifestations, thermoregulatory complaints, gastrointestinal symptoms, urinary frequency, body pain, and sleep disturbances. Long COVID (LCov) participants (*n* = 19, 14 female and 5 male) were selected with one or more of these symptoms, with moderate or worse severity, with onset less than three months following COVID-19 infection and persisting for at least three months according to the WHO working case definition^44^. Symptom severity is summarized in Table 1. Participants were not hospitalised and strains were not verified. LCov participants were aged between 30 and 65 years, with mean, SD = 48,13 years. Duration of illness was obtained via questionnaire. Healthy controls (*n* = 16, 11 female and 5 male) reported no chronic health condition or evidence of underlying illness. They may have had active COVID-19 infections but did not have sequelae lasting beyond convalescence. Healthy participants were aged between 22 and 60 years, with mean, SD = 39,13 years. Medical history was requested to identify comorbid manifestations or exclusionary diagnoses including mental illness, malignancies, autoimmune, neurological, or cardiovascular diseases. Female participants were not pregnant or breastfeeding. Recruitment was between 2022 and 2024.

### MRI acquisition

MRI images were acquired on a 7 Tesla whole-body MRI research scanner (Siemens Healthcare, Erlangen, Germany) with a 32-channel head coil (Nova Medical Wilmington, USA). The study acquired two 7.5 min fMRI separated by 90 s. Both were acquired sagitally for each subject during the cognitive Stroop color-word interference task. Data were not separated to congruent, incongruent and neutral stimulus responses for this analysis. The separate 7.5 min scans were a methodological convenience. The original intention was to repeat the 15 min BOLD scans of an earlier 3 T study^45^. The 7 T scanner software rejected this due to RF heating limits and offered two separate 7.5 min BOLD scans separated by 90 s instead. We accepted this compromise and used it throughout.

For each fMRI, 225 volumes of fMRI data were acquired using a multiband echo-planar imaging (EPI) pulse sequence developed at the University of Minnesota^46^, with 80 sagittal slices, multiband factor = 3, TR = 2000 ms, TE = 22.4 ms, flip angle = 70°, acquisition matrix 192 × 192 and voxel size 1.25 mm^3^. The 225 fMRI volumes were acquired while the participant responded to a sequence of Stroop colour-word tests^47^.

An anatomical image was acquired using Siemens T2 ‘SPACE’ optimized 3D fast spin-echo (T2wSE) TR = 3200ms, TE = 563ms, variable flip angle scans on an adjacent 3 T scanner on the same day. Acquisition time was 5:44 (min: sec). These scans employed an optimised variable flip angle sequence (Siemens SPACE) to yield a ‘true 3D’ acquisition in a shortened time. Their contrast compares with standard T2wSE^48^ although the signal is also influenced by T1 relaxation^49^, possibly more than for T2wSE. These T2 ‘SPACE’ images were sagittal with pixel size 0.88 × 0.88 × 0.9 mm. T2 ‘SPACE’ were chosen for the anatomical scan because their spin-echo sequence renders them resistant to the magnetic field induced distortions of the conventional gradient-echo MPRAGE sequences^50^, particularly in the brainstem.

### Cardiac and respiratory monitoring

Physiological noise in the fMRI BOLD time series was modelled using time series from pulse oximeter and respiratory strap sensors recorded during the scan. The cardiac and respiratory time series interactions were computed using the TAPAS toolbox^51^ and used as 18 covariates to isolate physiological noise.

### The stroop task

During each task fMRI, the colour-word Stroop task was used to investigate the attention and concentration difficulties reported by LCov patients^47^. Each Stroop task displayed two coloured words. The upper words were RED, BLUE, YELLOW, GREEN or XXXX and were coloured red, blue, yellow or green on a black background. The lower word was RED, BLUE, YELLOW or GREEN printed in white on a black background. The participants were asked to decide whether the *colour* of the upper word agreed with the *meaning* of the lower word and press one of two buttons on a handpiece to respond ‘yes’ or ‘no’. The confounding factor was introduced when the upper word did not match its colour.

The Stroop task fMRI was divided into four conditions, three were trials: *neutral* (upper word is XXXX), *congruent* (Congr) when the upper word ink was the same colour as the lower word’s meaning, and *incongruent* (Incon) when the colour of the upper word was a different from lower word’s meaning. The incongruent task is considered more challenging because an inhibitory element is required to overcome the natural impulse to read the upper word rather than inspect its colour in order to decide on meaning of the upper word vs. meaning of the lower word, rather than upper word colour vs. lower word meaning. The tendency for automatic reading of the upper word provides interference to responding to the colour identification task. The fourth condition, *rest*, was the period between a trial response and the next trial onset during which a fixed stationary cross appeared on the screen for a period randomised between 3 and 12 s.

In each task fMRI, a total of 60 Stroop trials were randomly distributed over each 7.5 min acquisition with average inter-stimulus time of 7.5s. 40% of trials were incongruent, 30% were congruent, and 30% were neutral. For each trial type, the stimulus-onset and response times were recorded, and the difference calculated as response time (RT).

Each participant was given a brief Stroop training session before commencement of the first 7T fMRI scan (Run 1). The subject was shown colour printouts of Stroop test screens that included examples of congruent, incongruent, and neutral trials and the correct responses explained.

Statistical inference p of RT differences between HC and LCov were estimated using the Excel ‘T.TEST’ function.

### MRI processing

All MRI data were processed using the CONN^52^ release 22.v2407 functional connectivity toolbox^53^ and SPM12^54^ toolbox.

Independent Component Analysis (ICA) of the BOLD time-series was performed to maximise sensitivity to connectivity differences between LCov and HC for each run. ICA aggregates the full spatial and temporal datasets of both groups and identifies independent components, each with a unique temporal and spatial signature, at the group and individual subject level. We chose 15 components as a compromise between the number of potential intrinsic networks vs. the complexity of their nodes and connections. A single independent component may include multiple spatially separated locations which, because they have a common temporal signature, can be regarded as functionally connected. ICA components were assigned to intrinsic networks based on their temporal correlation with CONN’s resting state template intrinsic networks.

### Preprocessing

Functional and anatomical data were preprocessed using CONN’s flexible preprocessing pipeline^53^ including removal of the first 5 fMRI volumes, realignment with correction of susceptibility distortion interactions, slice timing correction, outlier detection, direct segmentation and MNI-space normalisation, smoothing. Functional data were realigned using the SPM12 realign & unwarp procedure^55^, where all scans were coregistered to a reference (first) volume using a least squares approach and a 6 parameter (rigid body) transformation^56^, and resampled using b-spline interpolation to correct for motion and magnetic susceptibility interactions. Temporal misalignment between different slices of the functional data (acquired in interleaved Siemens order) was corrected following the SPM12 slice-timing correction (STC) procedure^57^ using sinc temporal interpolation to resample each slice BOLD timeseries to a common mid-acquisition time. Potential outlier scans were identified using ART as acquisitions with framewise displacement above 0.5 mm or global BOLD signal changes above 5 standard deviations^58^, and a reference BOLD image was computed for each participant by averaging all scans excluding outliers. Functional and anatomical data were normalised into standard MNI space, segmented into grey matter, white matter, and cerebral spinal fluid (CSF) tissue classes, and resampled to 1.25 mm isotropic voxels following a direct normalisation procedure^59^ using SPM12 unified segmentation and normalisation algorithm^60,61^ with the default IXI-549 tissue probability map template. Functional data were smoothed using spatial convolution with a Gaussian kernel of 5 mm full width half maximum (FWHM).

### Denoising

Functional time series were denoised using CONN’s standard denoising pipeline^53^ including the regression of potential confounding effects characterized by motion parameters and their first order derivatives (12 factors)^62^, outlier scans^58^, white matter timeseries (5 CompCor noise components), CSF timeseries (5 CompCor noise components), physio regressors (18 components), and linear trends (2 factors) within each functional run, followed by bandpass frequency filtering of the BOLD timeseries between 0.008 Hz and 0.09 Hz. CompCor^63^ noise components within white matter and CSF were estimated by computing the average BOLD signal as well as the largest principal components orthogonal to the BOLD average, motion parameters, and outlier scans within each subject’s eroded segmentation masks. From the number of noise terms included in this denoising strategy, the effective degrees of freedom of the BOLD signal after denoising were estimated to range from 50.5 to 58.4 (average 57.7) across all participants^64^.

### Independent component analysis (ICA)

Group-level ICA was performed within CONN’s First-level processing. The group-level components are first derived with the dedicated group algorithm of Calhoun et al^65^.. To derive the statistical inference of these group-level components, individual subject components are then computed from the group components using the GICA1 method described in Erhardt^66^. This facilitates a GLM analysis to derive the statistical inference Z of each group-level voxel. The two paragraphs below were extracted using CONN’s First-level processing ‘Methods’ tab.

#### First-level analysis:

Group-level independent component analyses (group-ICA^65^ were performed to estimate 15 temporally coherent networks from the fMRI data combined across all subjects. The BOLD signal from every timepoint and voxel in the brain was concatenated across subjects and conditions along the temporal dimension. A singular value decomposition of the z-score normalized BOLD signal (subject-level SVD) with 64 components separately for each subject was used as a subject-specific dimensionality reduction step. The dimensionality of the concatenated data was further reduced using a singular value decomposition (group-level SVD) with 15 components, and a fast-ICA fixed-point algorithm^67^ with hyperbolic tangent (G1) contrast function was used to identify spatially independent group-level networks from the resulting components. Last, GICA3 back-projection^66^was used to compute ICA maps associated with these same networks separately for each individual subject.

**Group-level analyses** were performed using a General Linear Model (GLM^53^. For each individual voxel a separate GLM was estimated, with first-level (individual subject) connectivity measures at this voxel as dependent variables, and group identifiers (and subject-level covariates) as independent variables. Voxel-level hypotheses were evaluated using multivariate parametric statistics with random-effects across subjects and sample covariance estimation across multiple measurements. Inferences were performed at the level of individual clusters (groups of contiguous voxels). Cluster-level inferences were based on parametric statistics from Gaussian Random Field theory^53,68^. Results were thresholded using a combination of a cluster-forming *p* < 0.001 voxel-level threshold, and a familywise corrected p-FDR < 0.05 cluster-size threshold^69^.

**Fig. 1:**
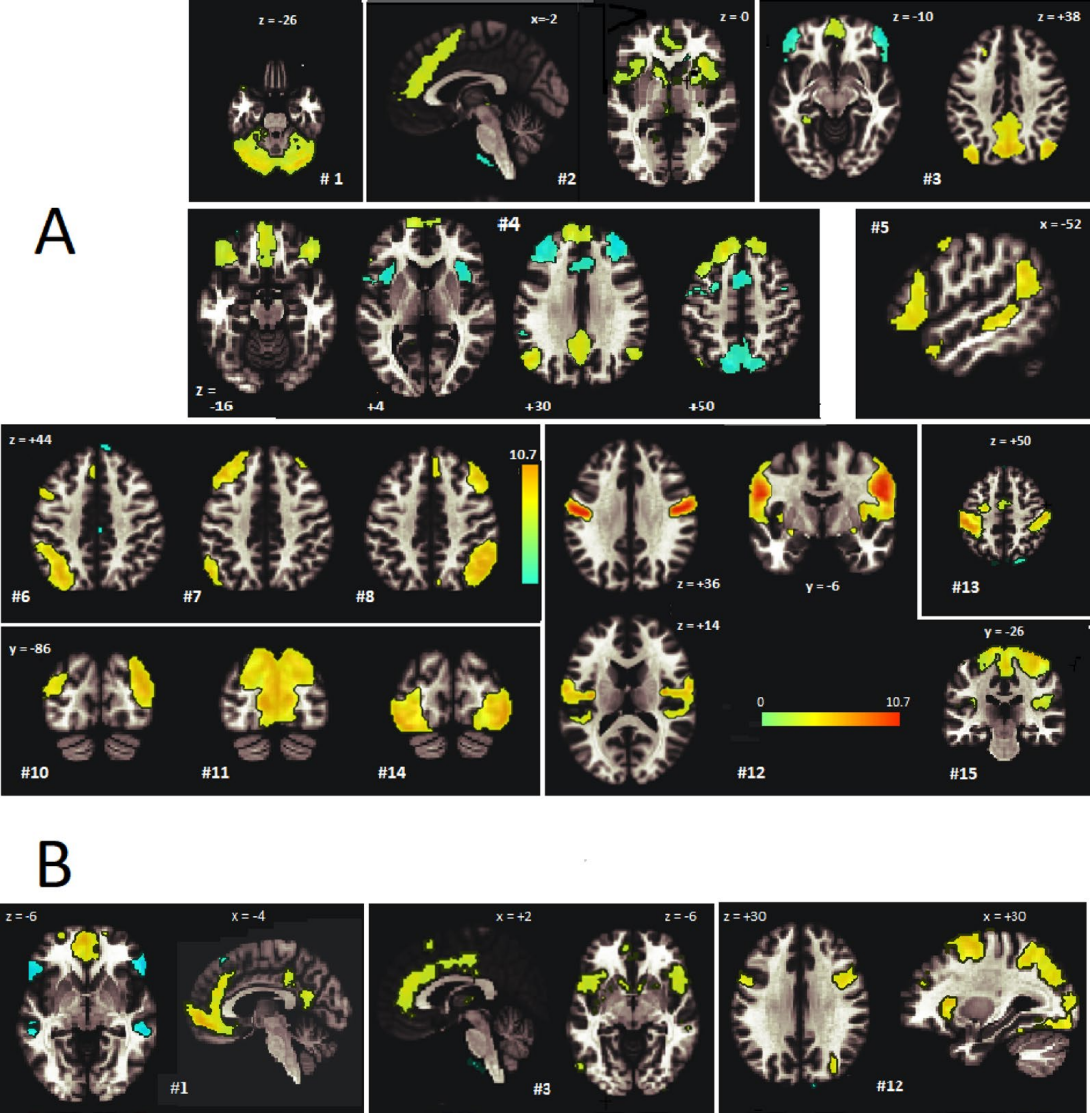
Sections through group-level ICA spatial maps for **(A)** 14 Run 1 components and **(B)** three Run 2 components. The component number is prefixed by #. Coloured voxels encode the statistical inference (Z) of the correlation coefficient of the group-level ICA component relative to that from GLM analysis of the individual subject ICA correlation coefficients at that voxel. Red-yellow voxels have significantly greater (via GLM analysis) and blue voxels significantly smaller group connectivity than the individual subjects. Single sections were chosen to highlight intrinsic network hubs. Their best fit networks were in A: #1 Cerebellar network; #2 Salience; #3 Default Mode network (DMN); #4 composite DMN, Central Executive (CE); #5 Language; #6, #7, #8 CE; #10, #11, #14 Visual; #12, #15 Sensorimotor; #13 Dorsal Attention. The Run 2 best fit in B was the Salience network for all 3 components shown. Other Run 2 ICA components occupied similar network locations to Run 1. Elevated connectivity is seen in the salience network bilateral insula and ACC hubs in both Runs (Run1 #2, Run 2 #3). Spatial maps are displayed with Z threshold = 2.5, except with a relaxed threshold = 2.0 for #1 #2 #4 in A; and for #3 in B.

This yields the voxel-wise statistical inference Z of each group-level component. This group Z statistic is color-coded onto the reference MRI in Fig. 1.

### ICA components and intrinsic networks

Multiple spatially separated areas of a single ICA component share the same temporal signature and can be said to exhibit connectivity. CONN numbered the ICA components according to their degree of overlap with grey matter. Each component was characterised as deriving from one (or more) of 8 intrinsic networks: Default Mode (DMN), Salience, Central Executive (CE also known as Fronto-Parietal), SensoriMotor, Language, Visual, Dorsal Attention or Cerebellar, according to its temporal signature regression with CONN’s resting-state network component temporal signature. Some ICA components involved multiple networks and some networks involved multiple ICA components. We show Z-maps for group ICA components associated with these intrinsic networks in Fig. 1.

### Inter-networks

Statistical analysis for detection of inter-networks was performed using a General Linear Model (GLM)^53^. For each individual voxel a separate GLM was estimated. The voxel GLM dependent variable was the vector of subject first-level connectivity measures (correlation coefficient between subject voxel BOLD and ICA component temporal signature). GLM independent variables were group identifiers and a subject-level covariate (age or illness duration). Voxel-level connectivity hypotheses, HC vs. LCov and LCov vs. illness duration, were evaluated using multivariate parametric statistics with random-effects across subjects and sample covariance estimation across multiple subjects. Inferences were performed at the level of individual clusters (groups of contiguous voxels). Cluster-level inferences were based on parametric statistics from Gaussian Random Field theory^53,68^. Results were thresholded using a combination of a cluster-forming *p* < 0.005 voxel-level threshold, and a family wise error (FWE) corrected p-FWE < 0.05 cluster-size threshold^69^. The relaxed 0.005 voxel-level threshold was chosen to detect significant clusters with enhanced sensitivity in this early 7 T MRI study of the challenging Long COVID illness.

Significant cluster to ICA component regressions constituted inter-network connectivity. The cluster was one hub of the inter-network, the ICA component spatial map the other.

Inter-network connectivity differences are assessed via the CONN ICA ‘RESULTS(2nd level)’ [spatial components] button. This permits selection of the binary contrast vectors for 16of35 HC and 19of35 LCov and the 35 ages. Between subjects contrast of 1–1 0 invokes GLM analysis of HC vs. LCov differences correcting for Age. Then selection of a single ICA component of interest and [display results] implements the whole brain voxel GLM (General Linear Model) analysis and displays whole brain maps of significant voxels and gives cluster details and statistics. These clusters are the areas where inter-network connectivity differs for HC and LCov.

Here we visualise voxel clusters of different LCov and HC inter-connectivity to each ICA component on surface views of the brain using semi-inflated white-matter surfaces. These views show the intersection of a cluster with the surface which sometimes causes a single cluster to appear as separated areas on the surface. We supplemented these images with clusters shown on axial sections of the SPM12 TPM reference grey matter brain.

## Results

### Long COVID symptom severity

Table 1 lists the frequency of occurrence of severity for eight Long COVID symptoms using six severity classes. Moderate severity was most common. Fatigue, headaches and unrefreshing sleep were mostly moderate or severe. Odour/taste, Sore throat or Muscle weakness were relatively uncommon.

**Table 1:** Symptom severity occurrence in 19 long COVID subjects for the severity levels: none, very mild, mild, moderate, severe or very severe.

Symptom	none	very mild	mild	moderate	severe	very severe	no data
Fatigue				11	8		
Concentration	1		4	7	6		1
Muscle ache	1	1	5	7	5		
Headaches	1		5	11	2		
Odour/taste	11		1	5			2
Unrefreshing sleep	2		1	7	8	1	
Sore throat	8	1	4	3	1	1	1
Muscle weakness	7	3	4	1	3		1

### Stroop task response times

For HC and LCov, Table 2 shows mean Response Times (RT) for each of the 3 Stroop task stimuli for both Run 1 and Run 2. LCov had significantly slower RT for all three stimuli in Run1 but only for InCon in Run2. Differences between runs were only significant for LCov (bottom row) for incongruent and congruent RTs. Accuracy was higher for HC but this was not significant (*p* > 0.05 not shown). RTs were faster in Run 2 than in Run 1, but with *p* < 0.05 only for Stroop stimuli Incon and Congr in LCov. Faster Run 2 RT indicates ‘Conflict Adaption’ or the ‘Practice Effect’. HC showed no significant change. Abnormal cognitive function in LCov was demonstrated by the prolonged RT at baseline (Run 1) and significant Run 2 adaption that was not seen in HC.

**Table 2:** Stroop task response times. Response times (RT) are compared between healthy controls (HC) and long COVID (LCov) for incongruent (Incon), congruent (Congr) and neutral Stroop stimuli during Run1 and Run2. Stroop Effect, (RT InCon – RT neutral)/(average RT) were also calculated. Statistical inference (*p*) is reported for between-group (HC vs. LCov) and between-Run tests (1 vs. 2, 2-sided) and values < 0.05 were regarded as significant. LCov showed significant Run2 RT reductions for incon and Congr stimuli.

cohort	Run	metric	Response Time (seconds)			Stroop
			InCon	Congr	Neutral	Effect
HC	1	mean	1.574	1.314	1.328	0.171
LCov	1	mean	2.053	1.794	1.648	0.145
HC vs. LCov	1	p	0.003	0.001	0.017	0.64
HC	2	mean	1.415	1.316	1.248	0.073
LCov	2	mean	1.699	1.526	1.476	0.102
HC vs. LCov	2	p	0.052	0.1	0.07	0.5
HC	1 vs. 2	p	0.3	1.0	0.50	0.055
LCov	1 vs. 2	p	0.015	0.049	0.19	0.4

Duration of illness was obtained via questionnaire and was between 0.16 and 3 years with median 0.50 years

### Independent component analysis (ICA)

Figure 1 (Table 3) illustrates ICA components from analysis of the aggregated HC and LCov cohorts. Their spatial extent maps the hubs of the brain’s intrinsic networks and demonstrates the multi-network activity stimulated by the Stroop task. Run 1 group-ICA components in Fig. 1A locate hubs of 14 intrinsic networks. The ICA voxel value displayed in colour is the Z statistic. Z = (X-µ)/σ where X is the group component correlation coefficient, and µ and σ are mean and standard deviation from General Linear Model (GLM) analysis of individual-subject correlation coefficients. The group-ICA intrinsic network(s) that CONN associated with each component are listed in the caption and in Tables 3 and 4. Their temporal correlation with CONN’s reference networks is listed in the ‘r’ column. Elevated connectivity is seen in the salience network bilateral insula and ACC hubs in both Runs (Run1 #2, Run 2 #3). Default Mode network (DMN, #3 in Fig. 1A) hubs included the PCC, L and R lateral parietal and medial prefrontal cortex. Fronto-parietal (Central Executive) hubs defined three independent networks (#6, #7, #8 in Fig. 1A), each iso-lateral in extent. Visual network activity also involved three independent networks (#10, #11, #14 in Fig. 1A) in bilateral areas of the occipital lobe with little overlap. ICA component #4 has partial spatial overlap of the DMN and CE networks although their temporal signature correlation with CONN’s reference networks was weak (*r* = 0.07 and 0.03). The sensorimotor network was seen in components #12 and #15. Tables 3 and 4 show r the temporal correlation of each ICA component with the resting state intrinsic network to which it was assigned by CONNexceeded 0.3 for all except the three (part-) Salience network components (Run 1 #2 and Run 2 #1, #3, #12), suggesting the salience network during the cognitive task differed so markedly from CONN’s reference resting state salience network that it resulted in allocation of salience to 3 ICA components.

Run 2 (Fig. 1B) had spatially similar ICA components to Run 1. It was notable that three Run 2 ICA components correlated with the reference Salience network, but only weakly (*r* = 0.08, 0.14, 0.09) for #1, #3 and #12. Run 2 ICA#1 and ICA#3 in Fig. 1B both involved the anterior cingulate cortex (ACC). Elevated connectivity is seen in the salience network bilateral insula and ACC hubs in both Runs (Run1 #2, Run 2 #3). Run 2 #12 involves bilateral precentral cortex (z = + 30) and right sided Insula (x = + 30), middle frontal cortex, superior parietal, and lateral occipital cortex (x = + 30). Thus, in Run 2, components #1, #3 and #12 all included known Salience network hubs while #12 involved diverse other hubs.

### Different HC vs. LCov internetwork connectivity

**Fig. 2:**
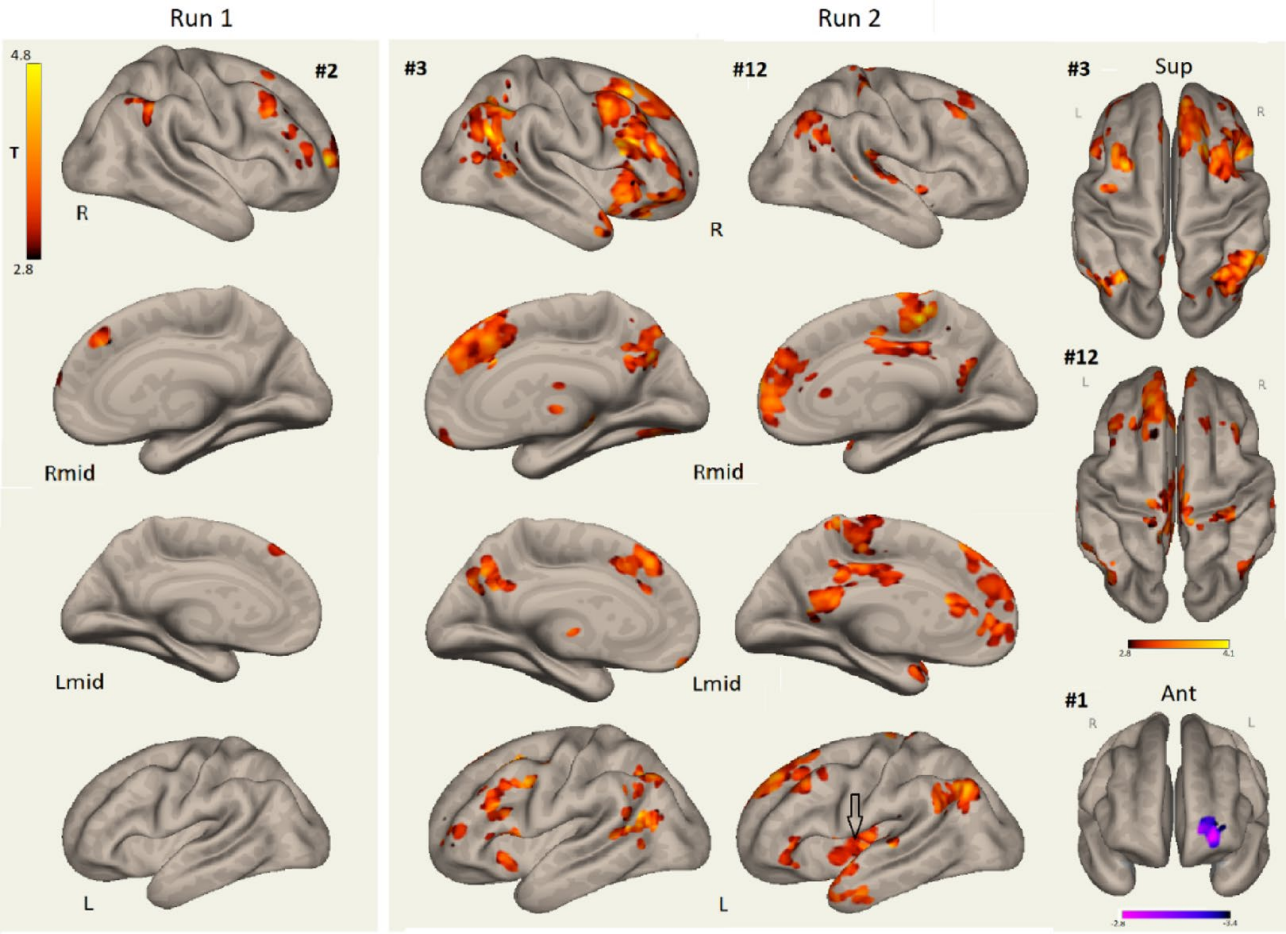
Salience network inter-network connectivity differences between HC and LCov in Run 1 (#2, Fig. 1A), and for overlapping Salience, Language and DMN networks in Run 2 (#1, #3, #12, Fig. 1B; Table 4). LCov connectivity deficits are shown in red-yellow and a single LCoV excess in blue (lower right). The arrow in the L view of Run 2 #12 locates the Broca’s language area. L is left, R is right and Lmid and Rmid are left and right midline views, Sup and Ant are superior and anterior views. Run 2 #3 clusters repeat the deficits of Run 1, but are deeper with stronger statistical inference and there are multiple additional deficits (details in Tables 3 and 4). The blue area of Salience component #1 (lower right) showed excess Run 2 LCov connectivity in the left frontal pole. 3D images were generated using CONN 22.v2407 www.nitrc.org/projects/conn.

**Fig. 3:**
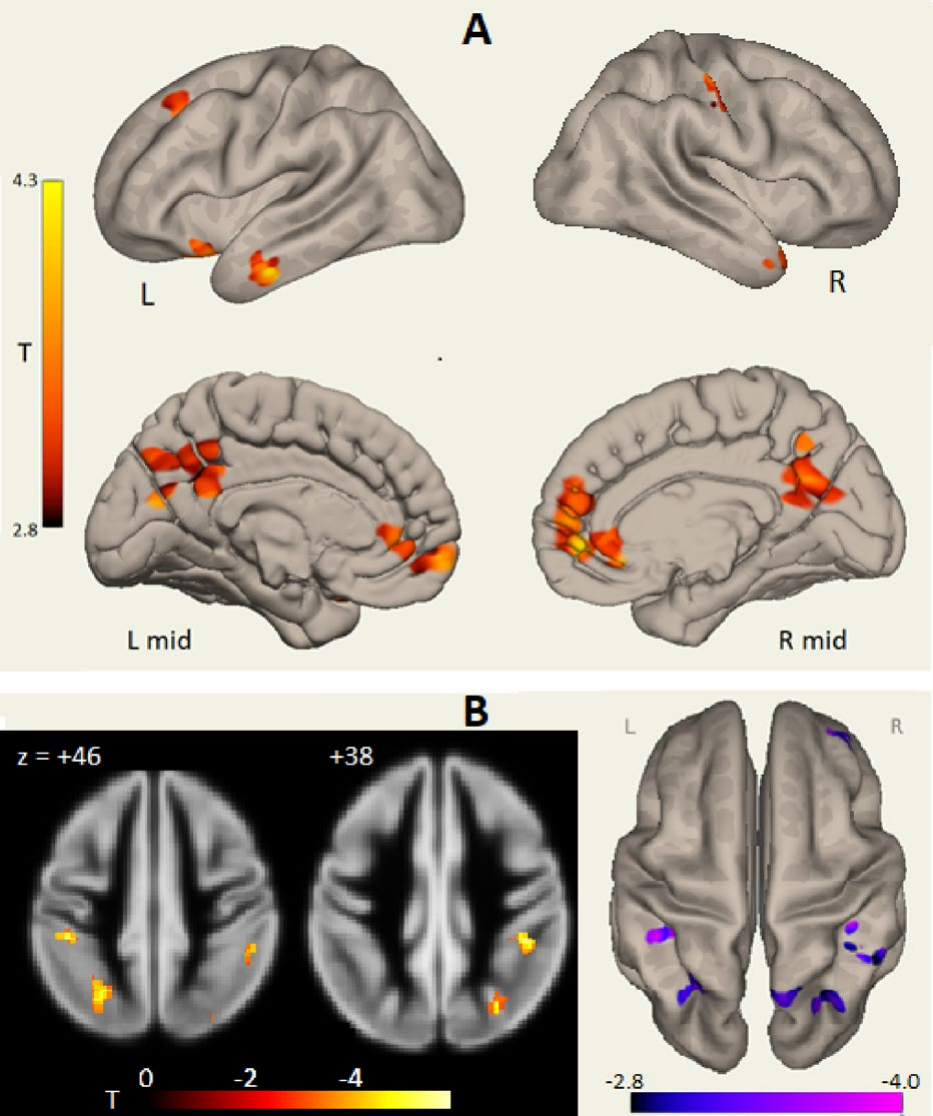
Run 1 Stroop-stimulated LCov connectivity deficits to Language network (ICA #5). Panel **A** locates LCov connectivity *deficits* on an inflated white matter surface (L and R) and on an inflated midline gray matter surface (L mid and R mid). Panel **B** shows *increased* LCov connectivity both on an inflated white matter surface (right, 4 clusters, two with separated surface crossings) and reference gray matter sections (lower left). In the sections, a cluster with increased LCov connectivity is shown at z = + 46 in the left superior lateral occipital lobe and at z = + 38 in the right supra marginal gyrus. Table 3 indicates their centres were at z = + 48 and z = + 44. Neighbouring clusters were not significant. See Table 3 for more details. In B, both colour bars encode negative T values (increased LCov connectivity). 3D images were generated using CONN 22.v2407 www.nitrc.org/projects/conn. Sections were generated by SPM12 https://www.fil.ion.ucl.ac.uk/spm/software/spm12/.

**Fig. 4:**
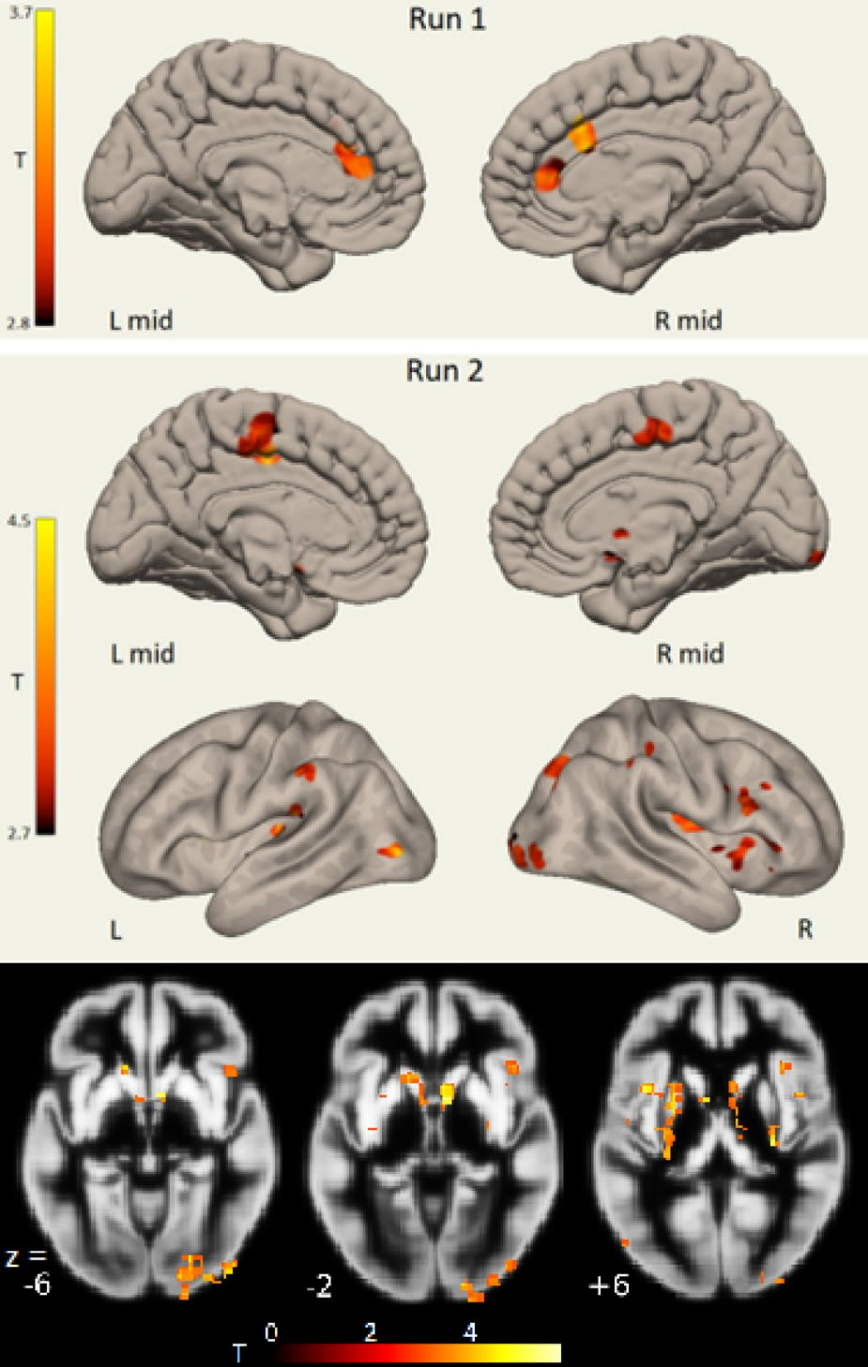
Central Executive (fronto-parietal) network LCov connectivity deficits in Run 1 (top) and Run 2 (below) shown intersecting inflated gray matter surfaces: see Table 3 ICA #8 and Table 4 ICA #9. L is left, R is right and *L mid* and *R mid* are left and right midline views. The Run 1 ACC connectivity deficit occurs bilaterally (same cluster) and R mid also shows a dorsal ACC deficit. Run 2 *L mid* and *R mid* views both show a contiguous cluster in the midline pre-central motor area. The highly significant right occipital pole deficits to CE (ICA #9) are located in the right (R) white matter surface and in sections at z= −6 and − 2. The basal ganglia (bilateral caudate and left putamen) LCov deficits reported in Table 4 are more clearly seen in the sections at z = −2 and + 6. 3D images were generated using CONN 22.v2407 www.nitrc.org/projects/conn. Sections were generated by SPM12 https://www.fil.ion.ucl.ac.uk/spm/software/spm12/.

**Fig. 5:**
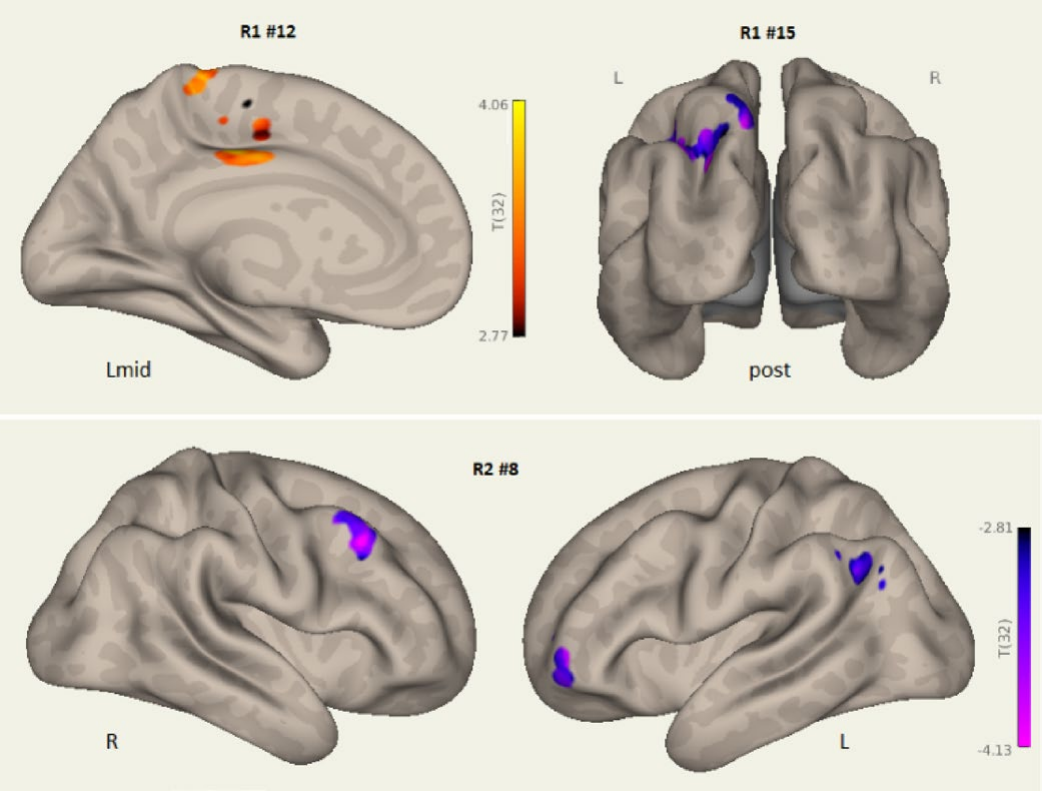
Sensorimotor network LCov connectivity deficits (red-yellow) and increased connectivity (blue) relative to HC to ICA components: Run1 #12, #15 and Run2 #8 (Tables 3 and 4 for details), all associated with network hubs. Views are inflated white matter surfaces from the midline of the left hemisphere (Lmid), posterior (post), right (R) and left (L). R1 is Run 1, R2 is Run 2. The Run 1 deficit in the posterior cingulate on the Lmid view had very high statistical inference (#12, *p* = 0.00008, Table 3). 3D images were generated using CONN 22.v2407 www.nitrc.org/projects/conn.

**Fig. 6:**
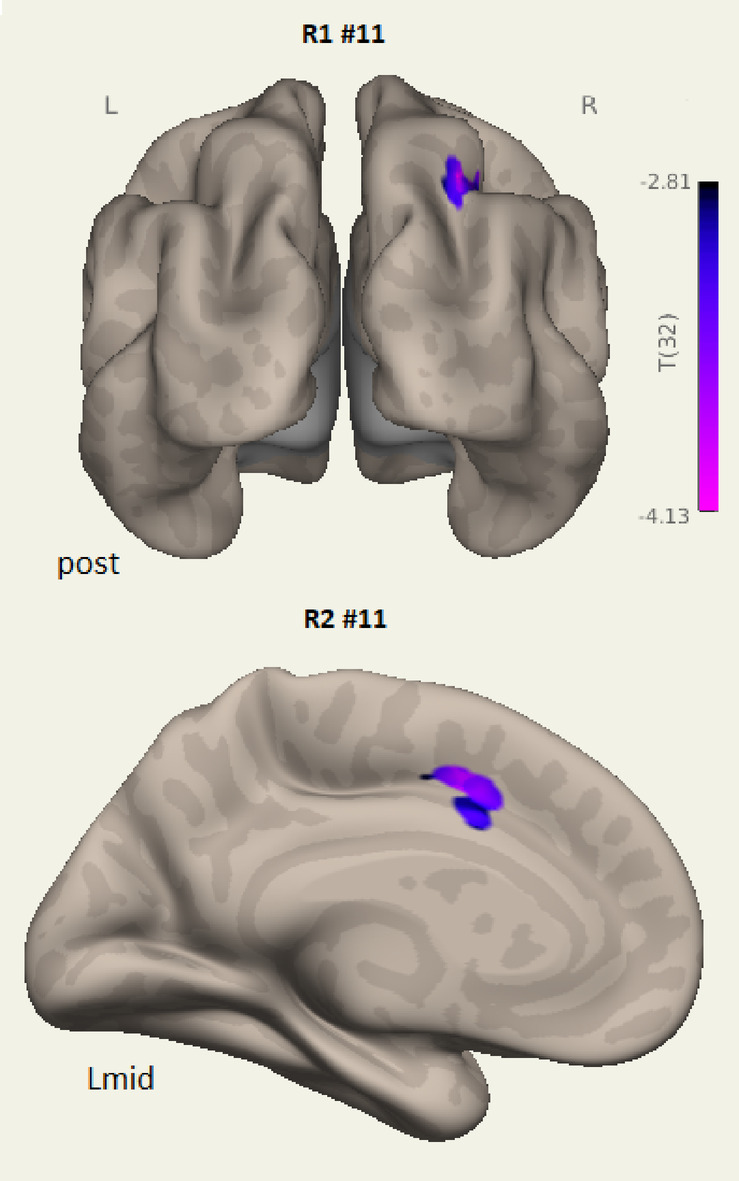
Visual network LCov increased connectivity excesses (blue) relative to HC to ICA components #11(Run 1) from the right lateral superior occipital/angular gyrus and #11(Run 2) from the bilateral dorsal ACC. Inflated white matter views are from the posterior (post) and left midline (Lmid). See Tables 3 and 4 for details. 3D images were generated using CONN 22.v2407 www.nitrc.org/projects/conn.

For each subject, for each ICA component, a spatial map of voxel connectivity to the ICA component (Fig. 1) was computed (First-level analysis, Methods) and represented ‘inter-network’ connectivity, the subject of this analysis. For each component, HC vs. LCov inter-network connectivity differences at the voxel level and voxel-cluster statistical inference were computed using a GLM model that isolated age effects. A significant cluster represented different HC and LCov connectivity between the cluster and the component’s network. Tables 3 and 4 list significant clusters with connectivity differences between LCov and HC for 5 components in Run 1 (Table 3) and 6 components in Run 2 (Table 4). These tables also list the intrinsic networks associated with each component by CONN and their % temporal correlation (not spatial overlap) with CONN’s template network. It is noteworthy that ICA components assigned solely or in part to the Salience network are distinguished by relatively weak correlations to CONN’s Salience network template (< 0.2), whereas all other networks have temporal correlations > 0.3. Two Run 2 Salience network ICA components (#3, #12) also correlated with reference DMN and Language networks. Different inter-network connections to these components showed very high statistical inference and feature in the Run 2 panel of Fig. 2B. Most differences were LCov connectivity deficits (low in LCov column), although three components in both Run1 and Run 2 showed *increased* connectivity in LCov relative to HC (‘high’ in LCov column). No inter-network connectivity differences were associated with DMN, Dorsal Attention or Cerebellar networks in either run. Locations in Figs. 2, 3, 4, 5 and 6 with different HC versus LCov connectivity to an intrinsic network were hubs with different ‘inter-network’ connectivity.

Temporal frequency and temporal variability did not differ between HC and LCov for any component for either run.

Figure 2 shows HC and LCov connectivity differences associated with the Salience network.

Impaired inter-network connectivity for Run2 component #3 involved frontal and lateral occipital areas. Run 2 LCov deficits were spatially larger than for the corresponding Run 1 component #2 and had stronger statistical inference (Tables 3 and 4). Inter-network connectivity deficits were also seen to Run 2 salience components #3 for language (see arrow for #12 L) and #12 for dorsolateral prefrontal and parietal angular gyrus cortices. Run 2 LCov connectivity to the Salience network (component #1) exceeded HC from the left frontal pole (Fig. 2 bottom right).

In Run 1 a language network LCov connectivity deficit to ICA component (#5 in Table 3; Fig. 1) was seen, while in Run 2 ICA #3 exhibited weaker LCov connectivity with Language, Salience and DMN networks (Fig. 1B; Table 4). Language network associated inter-network connectivity differences for Run 1 are shown in Fig. 3. The most significant deficits were from posterior DMN precuneus, and salience network paracingulate/ACC nodes (Fig. 3A L mid and R mid, Table 3). The ‘L’ view at top left in Fig. 3A shows LCov connectivity deficits near language areas in the orbitofrontal, middle temporal and superior frontal cortices. These areas were also seen in the Run 2 composite Salience/Language/DMN inter-network components (#3 and #12, Fig. 2L view). Figure 3B shows Run 1 *increased* LCov connectivity in the parietal association area/angular gyrus involving sensory and visual cortices on an inflated white matter surface (blue) and on two axial reference sections.

Clusters with LCov CE (central executive) network connectivity deficits are shown in Fig. 4. Run 1 and Run 2 deficits are quite different. Run 1 deficits from the ACC were seen in both L mid and R mid views (same cluster) with an separate dACC deficit in the Rmid view. Run 2 ICA#9 L mid and R mid views both show a connectivity deficit in the pre-central motor area. Run 2 deficits seen in the R view at the occipital pole (see also z = −6 section in the lower panel) and basal ganglia were highly significant (Table 4). The three basal ganglia clusters, listed in Table 4 as ‘Caudate L’, ‘Caudate R’ and ‘Putamen R’ were best seen in the sections at z = −2 and z = + 6.

Figure 5 locates differences in Stroop stimulated LCov connectivity to the Sensorimotor network in both Run 1 and Run 2. Clusters with both LCov connectivity deficits and excesses were detected, with LCov excesses more prevalent and in frontal and parietal association areas. Locations differed for Run 1 and Run 2 (#8) and from left to right in Run 2.

Figure 6 shows Stroop stimulated LCov connectivity excesses relative to HC associated with activity of a Visual network (component #11) in both runs. For Run 1 excess LCov connectivity was in the right angular gyrus and for Run 2 in the cingulate cortex.

**Table 3:** Run 1 connectivity differences to group ICA intrinsic networks for 16 HC vs. 19 LCov. Voxel clusters have p-FWE < 0.05. In the LCov column, ‘low’ indicates a LCov connectivity deficit relative to HC, ‘high’ LCov excess connectivity. The ‘r’ column is the ICA component Temporal correlation with conn’s intrinsic networks (network column). Unlisted networks did not differ in the HC and LCov connectivity to the rest of the brain. Cluster size is in voxels. Statistical inference p-FWE is based on cluster size. CE: central Executive, fron: frontal, SMC: supplementary motor cortex, SMG: Supra marginal gyrus, occip: occipital, ACC: anterior cingulate cortex, PCC: posterior cingulate cortex, lat: lateral, L: left, R: right, sup: superior, inf: inferior. Coordinates are for cluster centre, not most significant voxel.

ICA#	Network	*r*	LCov	Cluster centre	Cluster in	size	*p*-FWE
2	Salience	0.16	low	+ 42 +20 +40	Fron middle R	140	0.003
			low	+ 20 +60 +6	Fron pole R	125	0.006
			low	+ 48 −42 +44	SMG, Angular R	124	0.007
			low	+ 32 +16 +52	Fron middle sup R	114	0.011
5	Language	0.32	low	+ 2 −58 +32	precuneus	329	< 1e-6
			low	+ 6 +48 −4	paraCingulate	240	7e-6
			low	+ 6 +52 +16)	paraCingulate	130	0.002
			low	+ 54 −12 +48	Post-Central R	102	0.014
			low	−22 +32 +44)	Fron sup L	86	0.04
			**high**	−28 −64 +48	Occip Lat sup L	154	0.0006
			**high**	+ 46 −40 +44	SMG post R	106	0.01
8	CE	0.39	low	−2 +34 +16	ACC	94	0.024
			low	+ 4 +20 +30	dorsal ACC	83	0.05
11	Visual	0.61	**high**	+ 32 −60 +54	Occip Lat sup R	119	0.007
12	SensoriMotor	0.36	low	−2,−20,+42	PCC/SMC	192	0.00008
15	SensoriMotor	0.43	**high**	−16 −66 +50	Occip Lat sup L	162	0.003
			**high**	−40 −38 +42	SMG L	93	0.010

**Table 4:** Run 2 connectivity differences to ICA intrinsic networks for 16 HC vs. 19 LCov. Voxel clusters have p-FWE < 0.005. In the LCov column, ‘low’ indicates a LCov connectivity deficit relative to HC, ‘high’ LCov excess connectivity. The ‘r’ column is the ICA component Temporal correlation with conn’s reference networks (network column). Cluster size is in voxels. Statistical inference p-FWE is based on cluster size. See Table 3 caption for abbreviations. Note that coordinates are for cluster centre, not most significant voxel.

ICA#	network	*r*	LCov	Cluster centre	Cluster in	size	*p*-FWE
1	Salience	0.08	**high**	−24, + 60, −4	Fron pole L	139	0.002
3	Salience	0.14	low	+ 40 +16 +42	Fron middle R	2813	< 1e-6
	Language	0.12	low	+ 40 −66 +42	Occip Lat, Angular R	1528	< 1e-6
	DMN	0.05	low	+ 0 −66 +40	precuneus	450	< 1e-6
			low	−50 −68 +20	Occip Lat sup L	337	< 1e-6
			low	−32 +16 +52	Fron middle L	327	2e-6
			low	−46 +22 +28	Fron middle L	265	2e-5
			low	+ 34 +24 −8	Fron orbito R	216	0.0001
			low	−40 −66 +48	Occip Lat sup L	194	0.0004
			low	+ 40 +50 −10	Fron pole R	150	0.0003
			low	+ 12 + 16 + 6	Caudate R	120	0.02
			low	−30 +18 −6	Insula L	106	0.03
8	SensoriMotor	0.38	**high**	+ 42 +20 +44	Fron middle R	118	0.007
			**high**	−50 −64 +38	Occip Lat L	112	0.01
9	CE	0.37	low	+ 24 −94 −4	Occip pole R	237	7e-6
			low	+ 10 +10 +8	Caudate R	234	8e-6
			low	−24 −2 +6	Putamen L	228	1e-5
			low	+ 40 +22 +2	Fron operc R	164	0.0003
			low	−8 −22 +52	PreCentral	148	0.0008
			low	−8 +14 +0	Caudate L	125	0.003
			low	+ 44 + 14 + 26	Fron inf, operculumR	98	0.02
11	Visual	0.61	**high**	+ 4 + 10 + 38	ACC	150	0.001
12	Salience	0.09	low	−12,+52,+34	Fron pole L&R	1638	< 1e-6
	DMN	0.02	low	+ 2,−26,+42	PCC	685	< 1e-6
			low	−46,−68,+40	Occip Lat sup L	641	< 1e-6
			low	−46,+12,−18	Temporal pole L	444	< 1e-6
			low	−8,−24,+74	Pre, Post-Central	226	2e-5
			low	+ 44,−64,+44	Occip Lat, Angular R	210	0.00004
			low	−40,+22,+42	Fron middle L	160	0.0005
			low	−40,−4,+2	Fron inf, Insula L	160	0.0005
			low	+ 32,−30,+64	Post-Central R	150	0.0009
			low	−4,−48,+20	PCC	122	0.005
			low	+ 26,−70,−34	Cerebellum crus1,2 R	121	0.005
			low	−56,−2,−30	Temporal pole R	120	0.005

### Correlation with illness duration

**Fig. 7:**
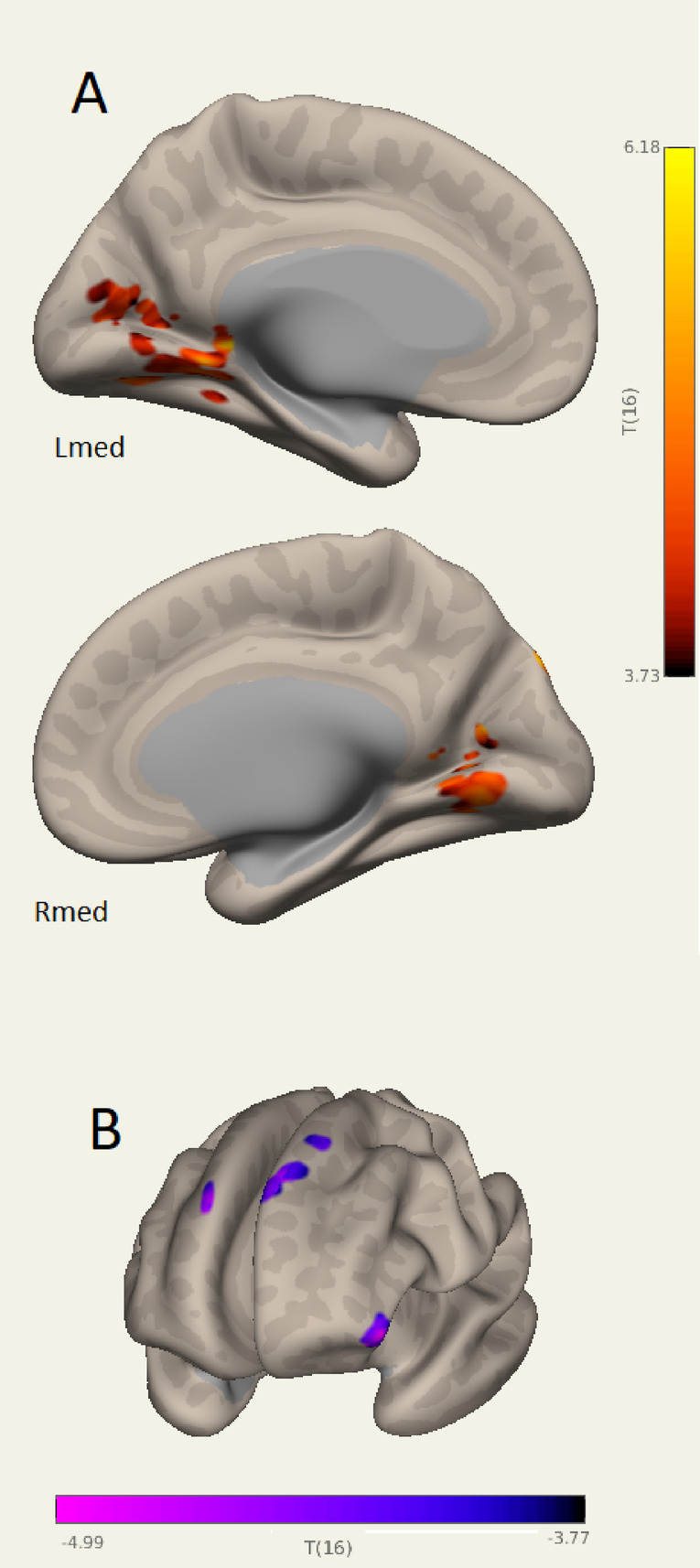
Run 1 clusters where network connectivity correlates with long COVID illness duration. (A) Lingual gyrus clusters with connectivity to Visual network (ICA #11 that correlates positively (strengthens) with long COVID duration with statistical inference (*p* < 1e-6, Table 5). Lmed and Rmed are the left medial and right medial views. (B) Fronto-superior view of frontal clusters with connectivity to the Salience network #2 which weakens with illness duration (T negative). The left frontal pole correlation has statistical inference *p* = 1e-6. The figure shows where each cluster crossed the semi inflated white matter surface. Single clusters appeared with multiple crossings. 3D images were generated using CONN 22.v2407 www.nitrc.org/projects/conn.

Statistical inference for inter-network correlations with illness duration is listed in Table 5. Negative correlations in the Table indicate connections to an intrinsic network which become weaker with increasing illness duration and include connections with the Salience, DMN and sensorimotor networks. Clusters with positive correlations have connections that become stronger with illness duration. Correlations were more prevalent in Run 1. Figure 7 shows example clusters where connectivity to a visual (ICA #11) network correlated with long COVID duration. Figure 7A shows the lingual cortex clusters for the Run1 Visual ICA#11 network with positive correlation (p-FWE < 1e-6). Figure 7B shows frontal clusters for the Salience ICA#2 network with negative correlations. Run 2 Visual network #11 coincides with Visual network #11 of Run 1 (Fig. 1A) although their inter-network correlations differ. The Salience network duration correlations, although involving a unilateral frontal pole in both runs, are on opposite sides and with opposite polarity for Run 1 and Run 2.

**Table 5:** Statistical inference for eight ICA components in run 1 and two in run 2 of their connectivity correlations with LCov illness duration elsewhere in the brain. In the ‘corr ‘ column ‘+’ indicates connectivity increased with illness duration and ‘-‘ that it decreased. Voxel clusters were formed for voxel p-FWE < 0.001. Cluster size k is number of voxels. Only clusters with p-FWE < 0.01 are listed. DMN: default mode network; CE: central executive (also known as fronto-parietal); DLPFC: dorsolateral prefrontal cortex; sensmot: sensori-motor network; SMG: Supra marginal gyrus.

ICA #	network	corr	Cluster centre	Cluster in	size k	*p*-FWE
**Run 1**						Pvoxel < 0.001
2	Salience	-	−44,+40,−8	Fron pole L	126	0.000001
		-	−10,+54,+40	Fron pole L	89	0.00003
		+	−48,−2,+42	precentral L	57	0.0006
3	DMN	+	−42,+4,+54	Fron middle	50	0.002
		+	−58,−48,+14	SMG Angular	46	0.003
		+	+ 4,+60,−16	Fron pole R	44	0.005
		-	+ 32,+32,+44	DLPFC R	111	0.000001
5	Language	+	−30,−98,−4	Occipital pole L	88	0.000008
		+	+ 10,−100,−10	Occipital pole R	52	0.001
6	CE	+	−46,−60,+10	Temporal middle	45	0.004
8	CE	+	+ 8,+56,+36	Fron pole R	55	0.001
9	CE	+	+ 8,+52,+44	Fron pole R	15	0.001
		+	−16,−58,−4	Lingual L	190	< 1e-6
11	Visual	+	+ 10,−66,0	Lingual R	157	< 1e-6
		+	−6,−78,+12	IntraCalcerine L	99	0.000005
		+	−18,−64,+54	Occip Lat L	48	0.003
12	SensMot	+	+ 46,−40,+48	SMG post R	41	0.009
		-	−12,−62,+20	Precuneus	102	0.000003
		-	+ 6,+52,+6	paraCing R	56	0.001
**Run 2**						
1	Salience	+	+ 48,+38,+20	Fron pole R	60	0.0004
		+	+ 42,+52,0	Fron pole R	41	0.007
11	Visual	-	−36,+40,+30	Fron pole L	90	0.00001
		-	−38,−82,+24	Occip Lat L	47	0.004

## Discussion

The Stroop colour-word task, which was performed during acquisition of each fMRI run, engaged all of the brain’s intrinsic networks. The stimulus response time (RT) provided an index of relative global brain performance^43^. LCov RT were slower than HC in both Run 1 and Run 2 confirming cognitive dysfunction. RT in HC were equivalent between runs while the faster LCov RT in Run2 which was significant for incongruent and congruent stimuli (Table 2) is consistent with conflict adaption^70^. The Stroop task, which exercised conflict detection and resolution, provoked slower brain response in LCov than HC although the faster LCov RT in Run 2 suggests conflict adaption.

The networks responsible for detecting and resolving task interference and motor execution were examined by ICA. ICA is a mathematical technique which processes a data entity including all corrected BOLD image time series from all subjects and identifies components with distinct spatial and temporal signatures. It was impressive that the spatial extent of the independent components coincided with the hubs of known intrinsic brain networks (Fig. 1) providing confidence that the fMRI data and the ICA technique were of adequate quality.

Despite CONN identifying several ICA components with the Salience network (#2 in Run 1 and #1, #3 and #12 in Run 2) their temporal correlations with CONN’s reference salience network were weak. Correlations for other networks were much stronger. This causes us to question whether CONN’s resting state salience network is useful for identifying networks associated with a cognitive task. It appears that Run 2 here may present a functionally distorted salience network. The dramatic Run 2 inter-network LCov connectivity deficits to the salience network ICA components #3 and #12 shown in Fig. 2 support a conclusion that salience network temporal signature distortions are propagated globally in LCov.

Run 2 started 90 s after the termination of the 450 s of Run (1) Fatigue induced by the cognitive exertion of Run 1 was anticipated to influence the cognitive performance in Run (2) The Run 2 Salience network was distributed over three ICA components (Fig. 1B; Table 4) two with overlapping DMN network areas. Run 2 ICA component #1 included lateral frontal and parietal regions with very small (blue in #1, Fig. 1B) group connectivity. Component #3 described anterior cingulate and anterior insula impairment which duplicated Run 1 #2 deficits. Component #12 was a complex of spatial signatures from insula, premotor, parietal and occipital nodes of networks that merged for the cognitive challenge of Run 2. Run 2 provided rich information on the impairments of LCov connectivity not present in Run 1 and suggests impairment of the salience network which monitors and preferentially excites Central Executive (FrontoParietal) or DMN activity^22^.

The three Run 2 Salience-network-associated components (Fig. 1B; Table 4) were exceptional because they exhibited widespread LCov connectivity deficits (Fig. 2). Deficits were much larger with stronger statistical inference for Run 2 than in congruent regions of Run 1. LCov deficits that were only seen for Run 2 suggest that a different response was activated in Run 2 involving more CE (fronto-parietal) hubs. LCov connectivity deficits in CE connectivity during Run 2 were more numerous and widely distributed than in Run 1 and incorporated sensorimotor and basal ganglia areas with very strong statistical inference (Fig. 4; Table 4). The increased magnitude of the deficits in LCov in Run 2 and creation of new networks with novel ICA temporal signatures and spatial extent overlapping several other networks (ICA #4 in Fig. 1A) together provides further evidence that Run 1 exacerbated cognitive dysfunction in Run 2 for LCov.

*Increased* LCov inter-network connectivity relative to HC was detected for Salience, Language, Sensorimotor and Visual networks (Figs. 2, 3, 5 and 6; Tables 3 and 4). Increases occurred in the angular gyrus (occipital lateral superior) area in three cases, and in the frontal pole in two. The angular gyrus integrates visual, motor and language inputs and responses, and its increased connectivity may represent a compensatory response to the widespread inter-network connectivity deficits elsewhere in the brain.

Sensorimotor and Language network associated internetwork connectivity deficits in both runs are consistent with diminished sensory input and/or lowered response in motor areas such as basal ganglia. The ACC detects conflict and engages DLPFC areas to modify task responses; It showed an activity deficit in Run 1 but not Run 2. These connectivity results are consistent with widespread brain function deficits in LCov which are stimulated by cognitive exertion.

Long Covid Connectivity became weaker with increasing post-infection illness duration for key internetwork connections, namely Salience to frontal pole and DMN to right dorsolateral prefrontal cortex (DLPFC). The DLPFC is activated for sustained and selective attention during task response. Various DLPFC nodes have different executive functions ranging from action selection and perception in the lateral frontal pole, affective valuation of effort and social cognition in the medial frontal pole, to language in Broca’s area of the left temporal pole. Thus, progressively reduced Salience to DLPFC connectivity is consistent with diminished functionality in long COVID.

Long Covid Connectivity became stronger with increasing post-infection illness duration for seven networks (Table 5, + in ‘corr’ column), namely the DMN, Language, three Central Executive (CE) networks, one Visual, and the Sensorimotor network. Such increases are consistent with compensatory connectivity in response to progressive impairment elsewhere.

Post-infection duration varied between nine months and three years. The median of 6 months shows most subjects had short duration of illness. Inclusion of more long illness duration subjects is desirable and may provide different insights.

Positive correlations with illness duration for the Visual (Run 1 #11 Fig. 7A) network to bilateral lingual gyri had very strong statistical inference. The lingual gyri are involved in visual processing of letters, encoding visual memories and analysis of logical conditions such as is required in the Stroop colour-word task and it appears processing of this task demands progressively stronger lingual connectivity with increasing LCov illness duration. The Stroop task also elicited positive correlation of LCov duration with Language network to Occipital pole connectivity. This is a verbal-visual connection for which the Stroop task also requires progressively stronger connectivity post-infection. The sensorimotor network (#12), which is involved in the motor action of button pressing during the Stroop task, exhibits duration correlation with the lateral occipital supra marginal gyrus regions which involve the angular gyrus, a region involved in language and number processing, memory and reasoning^71^. This connection becomes stronger with increasing LCov duration suggesting progressively enhanced connectivity in response to progressive impairment elsewhere.

We suggest that ongoing neuronal and glial fusogen action of SARS-Cov-2 virus in the brain^17^ may be the primary driver of LCov and that the widespread strengthening of connections with LCov progression may be a compensatory response to the progressive deficits of this infection.

The ICA technique here is shown to provide complex, in depth insights into the cerebral impairment associated with SARS-Cov-2 infection.

Although SF-36, WHODAS, and the Modified Fatigue Scale were completed, to limit manuscript length, we will present their correlations in a future publication.

### Limitations

Correlations with clinical parameters from neuropsychological testing in LCov could strengthen clinical relevance but were deferred to a subsequent publication. This small cross-sectional study provides correlational evidence in LCov but does not indicate causation. Stroop tests with errors were not removed which may affect RT means (Rabbitt effect)^72,73^ and networks that are activated during error processing^74^. Illustration of the location of LCov connectivity deficits and excesses using the intersection of voxel-clusters with semi-inflated white matter surfaces, while compact and intuitive, is limited when the intersection is small or zero. 2D representation of 3D results are necessarily limited. Identification of the intrinsic network associated with each ICA component was based on its temporal signature correlation with resting-state fMRI intrinsic networks from a CONN ICA analysis of healthy subjects. Weak correlation may result in mis-identification. This was problematic for our salience network analysis. Additional participants will be needed to assess longitudinal changes.

## Conclusions

Run 1 showed multiple regions with functional connectivity deficits in LCov compared to HC. The Run 1 Stroop cognitive exertion exacerbated these differences in Run 2 by inducing larger deficits for LCov and novel inter-network connectivity deficits with Salience and other networks. Duration dependence of LCov inter-network connectivity indicates progression of the disease with some compensatory changes. The widespread nature of LCov connectivity abnormalities supports a hypothesis that long COVID derives from brain-wide deficits. These may have their origin in COVID-19 damage to the whole brain such as from viral fusogens^17^ or specific brainstem damage^19,20,75–78^.

## Data Availability

The datasets generated and analysed during the current study are not publicly available due to Ethical constraints, but are available from the corresponding author on reasonable request.

## References

[1] Holmes, A. et al. Persistent symptoms after COVID-19: an Australian stratified random health survey on long COVID. *Med. J. Aust*. **221** (Suppl 9), S12–S17 (2024). 39489523 10.5694/mja2.52473

[2] Rofail, D. et al. Thematic analysis to explore patients’ experiences with long COVID-19: a conceptual model of symptoms and impacts on daily lives. *BMJ Open.* **14**, e076992 (2024). 38233059 10.1136/bmjopen-2023-076992 PMC10806796

[3] Thaweethai, T. et al. Development of a definition of postacute sequelae of SARS-CoV-2 infection. *JAMA* **329**, 1934–1946 (2023). 37278994 10.1001/jama.2023.8823 PMC10214179

[4] Baraniuk, J. N., Thapaliya, K., Inderyas, M., Shan, Z. Y. & Barnden, L. R. Stroop task and practice effects demonstrate cognitive dysfunction in long COVID and myalgic encephalomyelitis / chronic fatigue syndrome. *Sci. Rep.* **14**, 26796 (2024). 39500939 10.1038/s41598-024-75651-3 PMC11538523

[5] Chandan, J. S. et al. Non-Pharmacological therapies for Post-Viral Syndromes, including long COVID: A systematic review. *Int. J. Environ. Res. Public. Health*. **20**, 3477 (2023). 36834176 10.3390/ijerph20043477 PMC9967466

[6] Woo, M. S. et al. Frequent neurocognitive deficits after recovery from mild COVID-19. *Brain Commun.* **2**, fcaa205 (2020). 33376990 10.1093/braincomms/fcaa205 PMC7717144

[7] Hammerle, M. B. et al. Cognitive complaints assessment and neuropsychiatric disorders after mild COVID-19 infection. *Arch. Clin. Neuropsychol.* **38**, 196–204 (2023). 36464245 10.1093/arclin/acac093

[8] Henneghan, A. M., Lewis, K. A., Gill, E. & Kesler, S. R. Cognitive impairment in Non-critical, Mild-to-Moderate COVID-19 survivors. *Front. Psychol.* **13**, 770459 (2022). 35250714 10.3389/fpsyg.2022.770459 PMC8891805

[9] Hellmuth, J. et al. Persistent COVID-19-associated neurocognitive symptoms in non-hospitalized patients. *J. Neurovirol*. **27**, 191–195 (2021). 33528824 10.1007/s13365-021-00954-4 PMC7852463

[10] Panagea, E. et al. Neurocognitive impairment in long COVID: A systematic review. *Arch. Clin. Neuropsychol.* **40**, 125–149 (2024). 10.1093/arclin/acae042 PMC11754669 38850628

[11] González-Rosa, J. J. et al. Structural and functional brain markers of cognitive impairment in healthcare workers following mild SARS-CoV-2 infection during the original stream. *Brain Commun.* **6**, fcae340 (2024). 39416878 10.1093/braincomms/fcae340 PMC11481020

[12] Zhao, S. et al. Long COVID is associated with severe cognitive slowing: a multicentre cross-sectional study. *eClinicalMedicine* **68**, 102434 (2024). 38318123 10.1016/j.eclinm.2024.102434 PMC10839583

[13] Leitner, M., Pinter, D., Ropele, S. & Koini, M. Functional connectivity changes in long-Covid patients with and without cognitive impairment. *Cortex* **191**, 74–89 (2025). 40780102 10.1016/j.cortex.2025.07.005

[14] Douaud, G. et al. SARS-CoV-2 is associated with changes in brain structure in UK biobank. *Nature.* **604**, 697-707 (2022). 10.1038/s41586-022-04569-5 PMC9046077 35255491

[15] Ajčević, M. et al. Cerebral hypoperfusion in post-COVID-19 cognitively impaired subjects revealed by arterial spin labeling MRI. *Sci. Rep.* **13**, 5808 (2023). 37037833 10.1038/s41598-023-32275-3 PMC10086005

[16] Alhazmi, F. H. et al. Identifying cerebral microstructural changes in patients with COVID-19 using MRI: A systematic review. *Brain Circ.* **9**, 6–15 (2023). 37151797 10.4103/bc.bc_77_22 PMC10158661

[17] Martínez-Mármol, R. et al. SARS-CoV-2 infection and viral fusogens cause neuronal and glial fusion that compromises neuronal activity. *Sci. Adv.* **9**, eadg2248 (2023). 37285437 10.1126/sciadv.adg2248 PMC10246911

[18] Kesler, S. R. et al. Altered functional brain connectivity, efficiency, and information flow associated with brain fog after mild to moderate COVID-19 infection. *Sci. Rep.* **14**, 22094 (2024). 39333726 10.1038/s41598-024-73311-0 PMC11437042

[19] Barnden, L., Thapaliya, K., Eaton-Fitch, N., Barth, M. & Marshall-Gradisnik, S. Altered brain connectivity in long Covid during cognitive exertion: a pilot study. *Front. Neurosci.* **17**, 1182607 (2023). 37425014 10.3389/fnins.2023.1182607 PMC10323677

[20] Thapaliya, K., Marshall-Gradisnik, S., Barth, M., Eaton-Fitch, N. & Barnden, L. Brainstem volume changes in myalgic encephalomyelitis/chronic fatigue syndrome and long COVID patients. *Front. Neurosci.* **17**, 1125208 (2023). 36937672 10.3389/fnins.2023.1125208 PMC10017877

[21] Yeo, B. T. T. et al. The organization of the human cerebral cortex estimated by intrinsic functional connectivity. *J. Neurophysiol.* **106**, 1125–1165 (2011). 21653723 10.1152/jn.00338.2011 PMC3174820

[22] Menon, V. Large-scale brain networks and psychopathology: a unifying triple network model. *Trends Cogn. Sci.* **15**, 483–506 (2011). 21908230 10.1016/j.tics.2011.08.003

[23] Menon, V. & Uddin, L. Q. Saliency, switching, attention and control: a network model of Insula function. *Brain Struct. Funct.* **214**, 655–667 (2010). 20512370 10.1007/s00429-010-0262-0 PMC2899886

[24] Aboud, K. S. et al. Structural covariance across the lifespan: brain development and aging through the lens of inter-network relationships. *Hum. Brain Mapp.* **40**, 125–136 (2018). 30368995 10.1002/hbm.24359 PMC6478172

[25] Rong, B. et al. Widespread Intra- and Inter-Network dysconnectivity among Large-Scale resting state networks in schizophrenia. *J. Clin. Med.* **12**, 3176 (2023). 37176617 10.3390/jcm12093176 PMC10179370

[26] Li, W. et al. Intrinsic inter-network brain dysfunction correlates with symptom dimensions in late-life depression. *J. Psychiatr Res.* **87**, 71–80 (2017). 28017917 10.1016/j.jpsychires.2016.12.011 PMC5336398

[27] Metwali, H., Raemaekers, M., Ibrahim, T. & Samii, A. Inter-Network functional connectivity changes in patients with brain tumors: A Resting-State functional magnetic resonance imaging study. *World Neurosurg.* **138**, e66–e71 (2020). 32014546 10.1016/j.wneu.2020.01.177

[28] Stroop, J. R. Studies of interference in serial verbal reactions. *J. Exp. Psychol.* **18**, 643 (1935).

[29] Periáñez, J. A., Lubrini, G., García-Gutiérrez, A. & Ríos-Lago, M. Construct validity of the Stroop Color-Word test: influence of speed of visual Search, verbal Fluency, working Memory, cognitive Flexibility, and conflict monitoring. *Arch. Clin. Neuropsychol.* **36**, 99–111 (2021). 32514527 10.1093/arclin/acaa034

[30] Kalanthroff, E. & Henik, A. Preparation time modulates pro-active control and enhances task conflict in task switching. *Psychol. Res.* **78**, 276–288 (2014). 23712333 10.1007/s00426-013-0495-7

[31] Perrotta, D., Bianco, V., Berchicci, M., Quinzi, F. & Perri, R. L. Anodal tDCS over the dorsolateral prefrontal cortex reduces Stroop errors. A comparison of different tasks and designs. *Behav. Brain Res.* **405**, 113215 (2021). 33662440 10.1016/j.bbr.2021.113215

[32] Banich, M. T. The Stroop effect occurs at multiple points along a cascade of control: evidence from cognitive neuroscience approaches. *Frontiers Psychology.* **10**, 2164 (2019). 10.3389/fpsyg.2019.02164 PMC6797819 31681058

[33] Bush, G. et al. The counting stroop: an interference task specialized for functional neuroimaging–validation study with functional MRI. *Hum. Brain Mapp.* **6**, 270–282 (1998). 9704265 10.1002/(SICI)1097-0193(1998)6:4<270::AID-HBM6>3.0.CO;2-0 PMC6873370

[34] Banich, M. T. et al. fMRI studies of Stroop tasks reveal unique roles of anterior and posterior brain systems in attentional selection. *J. Cogn. Neurosci.* **12**, 988–1000 (2000). 11177419 10.1162/08989290051137521

[35] Milham, M. P., Banich, M. T., Claus, E. D. & Cohen, N. J. Practice-related effects demonstrate complementary roles of anterior cingulate and prefrontal cortices in attentional control☆☆This study was supported by the Beckman Institute for advanced science and technology at the university of Illinois, Urbana-Champaign; Carle Clinic, Urbana, Illinois; and NIMH MD/PhD predoctoral National research service award provided support to M.P.M. (MH12415-01). *NeuroImage* **18**, 483–493 (2003). 12595201 10.1016/s1053-8119(02)00050-2

[36] Carter, C. S. et al. Parsing executive processes: strategic vs. evaluative functions of the anterior cingulate cortex. *Proc. Natl. Acad. Sci. U S A*. **97**, 1944–1948 (2000). 10677559 10.1073/pnas.97.4.1944 PMC26541

[37] Pardo, J. P., et al.. The anterior cingulate cortex mediates processing selection in the Stroop attentional conflict paradigm. *Proceedings Natl. Acad. Sci. United States America* **87**, 256-259 (1990). 10.1073/pnas.87.1.256 PMC53241 2296583

[38] Gruber, S. A., Rogowska, J., Holcomb, P. & Soraci, S. Yurgelun-Todd, D. Stroop performance in normal control subjects: an fMRI study. *Neuroimage* **16**, 349–360 (2002). 12030821 10.1006/nimg.2002.1089

[39] Bianco, V. et al. Electrophysiological evidence of anticipatory cognitive control in the Stroop task. *Brain Sci.* **11**, 783 (2021). 34199201 10.3390/brainsci11060783 PMC8231961

[40] Ghahremani, A., Rastogi, A. & Lam, S. The role of right anterior Insula and salience processing in inhibitory control. *J. Neurosci.* **35**, 3291–3292 (2015). 25716829 10.1523/JNEUROSCI.5239-14.2015 PMC6605563

[41] Aron, A. R., Robbins, T. W. & Poldrack, R. A. Inhibition and the right inferior frontal cortex: one decade on. *Trends Cogn. Sci.* **18**, 177–185 (2014). 24440116 10.1016/j.tics.2013.12.003

[42] Bari, A. & Robbins, T. W. Inhibition and impulsivity: Behavioral and neural basis of response control. *Progress Neurobiology.* **108**, 44-79 (2013). 10.1016/j.pneurobio.2013.06.005 23856628

[43] Deng, Y., Wang, X., Wang, Y. & Zhou, C. Neural correlates of interference resolution in the multi-source interference task: a meta-analysis of functional neuroimaging studies. *Behav. Brain Funct.* **14**, 8 (2018). 29636070 10.1186/s12993-018-0140-0 PMC5891971

[44] Soriano, J. B., Murthy, S., Marshall, J. C., Relan, P. & Diaz, J. V. A clinical case definition of post-COVID-19 condition by a Delphi consensus. *Lancet Infect. Dis.* **22**, e102–e107 (2022). 34951953 10.1016/S1473-3099(21)00703-9 PMC8691845

[45] Shan, Z. Y. et al. Decreased connectivity and increased blood oxygenation level dependent complexity in the default mode network in individuals with chronic fatigue syndrome. *Brain Connect.* **8**, 33–39 (2018). 29152994 10.1089/brain.2017.0549

[46] Auerbach, E. J., Xu, J., Yacoub, E., Moeller, S. & Ugurbil, K. Multiband accelerated spin-echo echo planar imaging with reduced peak RF power using time-shifted RF pulses. *Magn. Reson. Med.* **69**, 1261–1267 (2013). 23468087 10.1002/mrm.24719 PMC3769699

[47] Leung, H. C., Skudlarski, P., Gatenby, J. C., Peterson, B. S. & Gore, J. C. An event-related functional MRI study of the stroop color word interference task. *Cerebral cortex (New York, N.Y.*) **10**, 552–60 (2000).) 10, 552–60 (2000). (1991). 10.1093/cercor/10.6.552 10859133

[48] Busse, R. F., Hariharan, H., Vu, A. & Brittain, J. H. Fast spin echo sequences with very long echo trains: design of variable refocusing flip angle schedules and generation of clinical T2 contrast. *Magn. Reson. Med.* **55**, 1030–1037 (2006). 16598719 10.1002/mrm.20863

[49] Mugler, J. P. Optimized three-dimensional fast-spin-echo MRI. *J. Magn. Reson. Imaging*. **39**, 745–767 (2014). 24399498 10.1002/jmri.24542

[50] Bushberg, J. T., Seibert, J. A., Leidholdt, E. M. & Boone, J. M. *The Essential Physics of Medical Imaging* (Lippincott, Williams and Wilkins, 2002).

[51] Kasper, L. et al. The physio toolbox for modeling physiological noise in fMRI data. *J. Neurosci. Methods*. **276**, 56–72 (2017). 27832957 10.1016/j.jneumeth.2016.10.019

[52] Whitfield-Gabrieli, S. & Nieto-Castanon, A. Conn: a functional connectivity toolbox for correlated and anticorrelated brain networks. *Brain Connect.* **2**, 125–141 (2012). 22642651 10.1089/brain.2012.0073

[53] Nieto-Castanon, A. *Handbook of Functional Connectivity Magnetic Resonance Imaging Methods in CONN* (Hilbert, 2020).

[54] Penny, W. D., Friston, K. J., Ashburner, J. T., Kiebel, S. J. & Nichols, T. E. *Statistical Parametric Mapping: the Analysis of Functional Brain Images* (Elsevier, 2011).

[55] Andersson, J. L., Hutton, C., Ashburner, J., Turner, R. & Friston, K. Modeling geometric deformations in EPI time series. *Neuroimage* **13**, 903–919 (2001). 11304086 10.1006/nimg.2001.0746

[56] Friston, K. J. et al. Spatial registration and normalization of images. *Hum. Brain. Mapp.* **3**, 165–189 (1995).

[57] Sladky, R. et al. Slice-timing effects and their correction in functional MRI. *Neuroimage* **58**, 588–594 (2011). 21757015 10.1016/j.neuroimage.2011.06.078 PMC3167249

[58] Power, J. D. et al. Methods to detect, characterize, and remove motion artifact in resting state fMRI. *Neuroimage* **84**10.1016/j.neuroimage.2013.08.048 (2014). 10.1016/j.neuroimage.2013.08.048 PMC3849338 23994314

[59] Calhoun, V. D. et al. The impact of T1 versus EPI Spatial normalization templates for fMRI data analyses. *Hum. Brain Mapp.* **38**, 5331–5342 (2017). 28745021 10.1002/hbm.23737 PMC5565844

[60] Ashburner, J. & Friston, K. J. Unified segmentation. *NeuroImage.* **26**, 839–51 (2005). 10.1016/j.neuroimage.2005.02.018 15955494

[61] Ashburner, J. A fast diffeomorphic image registration algorithm. *Neuroimage* **38**, 95–113 (2007). 17761438 10.1016/j.neuroimage.2007.07.007

[62] Friston, K. J., Williams, S., Howard, R., Frackowiak, R. S. & Turner, R. Movement-related effects in fMRI time-series. *Magn. Reson. Med.* **35**, 346–355 (1996). 8699946 10.1002/mrm.1910350312

[63] Chai, X. J., Castañón, A. N., Ongür, D. & Whitfield-Gabrieli, S. Anticorrelations in resting state networks without global signal regression. *Neuroimage* **59**, 1420–1428 (2012). 21889994 10.1016/j.neuroimage.2011.08.048 PMC3230748

[64] Nieto-Castanon, A. Preparing fMRI Data for Statistical Analysis. Preprint at (2022). 10.48550/arXiv.2210.13564

[65] Calhoun, V. D., Adali, T., Pearlson, G. D. & Pekar, J. J. A method for making group inferences from functional MRI data using independent component analysis. *Hum. Brain Mapp.* **14**, 140–151 (2001). 11559959 10.1002/hbm.1048 PMC6871952

[66] Erhardt, E. B. et al. Comparison of multi-subject ICA methods for analysis of fMRI data. *Hum. Brain Mapp.* **32**, 2075–2095 (2011). 21162045 10.1002/hbm.21170 PMC3117074

[67] Hyvärinen, A. Fast and robust fixed-point algorithms for independent component analysis. *IEEE Trans. Neural Netw.* **10**, 626–634 (1999). 18252563 10.1109/72.761722

[68] Worsley, K. J. et al. A unified statistical approach for determining significant signals in images of cerebral activation. *Hum. Brain Mapp.* **4**, 58–73 (1996). 20408186 10.1002/(SICI)1097-0193(1996)4:1<58::AID-HBM4>3.0.CO;2-O

[69] Chumbley, J., Worsley, K., Flandin, G. & Friston, K. Topological FDR for neuroimaging. *Neuroimage* **49**, 3057–3064 (2010). 19944173 10.1016/j.neuroimage.2009.10.090 PMC3221040

[70] Egner, T. & Hirsch, J. The neural correlates and functional integration of cognitive control in a Stroop task. *NeuroImage* **24**, 539–547 (2005). 15627596 10.1016/j.neuroimage.2004.09.007

[71] Moreno, R. A. & Holodny, A. I. Functional brain anatomy. *Neuroimaging Clin. N. Am.* **31**, 33–51 (2021). 33220827 10.1016/j.nic.2020.09.008

[72] Rabbitt, P. M. Errors and error correction in choice-response tasks. *J. Exp. Psychol.* **71**, 264–272 (1966). 5948188 10.1037/h0022853

[73] Pfister, R. & Foerster, A. How to measure post-error slowing: the case of pre-error speeding. *Behav. Res. Methods*. **54**, 435–443 (2022). 34240334 10.3758/s13428-021-01631-4 PMC8863758

[74] Cieslik, E. C., Ullsperger, M., Gell, M., Eickhoff, S. B. & Langner, R. Success versus failure in cognitive control: Meta-analytic evidence from neuroimaging studies on error processing. *Neurosci. Biobehav Rev.* **156**, 105468 (2024). 37979735 10.1016/j.neubiorev.2023.105468 PMC10976187

[75] Hugon, J. et al. Cognitive decline and brainstem hypometabolism in long COVID: A case series. *Brain Behav.* **12**, e2513 (2022). 35290729 10.1002/brb3.2513 PMC9014998

[76] Richter, D. et al. Hypoechogenicity of brainstem Raphe in long-COVID syndrome-less common but independently associated with depressive symptoms: a cross-sectional study. *J. Neurol.* **269**, 4604–4610 (2022). 35552501 10.1007/s00415-022-11154-3 PMC9098142

[77] Stein, J. A., Kaes, M., Smola, S. & Schulz-Schaeffer, W. J. Neuropathology in COVID-19 autopsies is defined by microglial activation and lesions of the white matter with emphasis in cerebellar and brain stem areas. *Front. Neurol.* **14**, 1229641 (2023). 37521293 10.3389/fneur.2023.1229641 PMC10374362

[78] Kamasak, B. et al. Effects of COVID-19 on brain and cerebellum: a voxel based morphometrical analysis. *Bratisl Lek Listy*. **124**, 442–448 (2023). 36876379 10.4149/BLL_2023_068

